# Analyses of Clinical and Biological Data for French and Belgian Immunocompetent Patients Infected With Hepatitis E Virus Genotypes 4 and 3

**DOI:** 10.3389/fmicb.2021.645020

**Published:** 2021-04-14

**Authors:** Florence Micas, Vanessa Suin, Jean-Marie Péron, Caroline Scholtes, Edouard Tuaillon, Thomas Vanwolleghem, Laurence Bocket, Sébastien Lhomme, Chloé Dimeglio, Jacques Izopet, Florence Abravanel

**Affiliations:** ^1^Virology Laboratory, National Reference Centre of Hepatitis E Viruses, Federal Institute of Biology, University Hospital Center, Toulouse, France; ^2^National Reference Centre of Hepatitis Viruses, Sciensano, Brussels, Belgium; ^3^Department of Gastroenterology, Rangueil University Hospital, Toulouse, France; ^4^INSERM U1052-Cancer Research Center of Lyon (CRCL), Lyon, France; ^5^University of Lyon, University Claude Bernard Lyon 1 (UCBL1), Lyon, France; ^6^Department of Virology, Croix Rousse Hospital, Hospices Civils de Lyon, Lyon, France; ^7^Pathogenesis and Control of Chronic Infections, INSERM, University of Montpellier, Etablissement Français du Sang, CHU Montpellier, Montpellier, France; ^8^Department of Gastroenterology and Hepatology, Antwerp University Hospital, Edegem, Belgium; ^9^Laboratory of Experimental Medicine and Pediatrics, University of Antwerp, Antwerp, Belgium; ^10^Virology Laboratory EA3610, Faculty of Medicine, University Hospital Center, Lille, France; ^11^UMR Inserm U1043, UMR CNRS, U5282, Centre de Physiopathologie de Toulouse Purpan, Toulouse, France

**Keywords:** hepatitis E virus, genotype 4, pathogenicity, immunocompetent, Europe, acute hepatitis

## Abstract

Hepatitis E virus (HEV) genotypes 3 and 4 are the major causes of acute hepatitis in industrialized countries. Genotype 3 is mainly found in Europe and America, while genotype 4 is predominant in Asia. Several Japanese studies have suggested that genotype 4 is more virulent than genotype 3. We investigated this aspect by analyzing the clinical and biological data for 27 French and Belgian immunocompetent patients infected with HEV genotype 4. Their infections were probably acquired locally, since none of these patients reported traveling outside France or Belgium during the 2–8 weeks before symptoms onset. Each patient was matched for age (±5 years) and gender with two patients infected with HEV genotype 3. Bivariate analysis indicated that the HEV genotype 4-infected patients had significantly higher alanine aminotransferase (ALT) (2067 IU/L) and aspartate aminotransferase (AST) (1581 IU/L) activities and total bilirubin concentrations (92.4 μmol/L) than did those infected with HEV genotype 3 (1566 IU/L, *p* = 0.016; 657 IU/L, *p* = 0.003 and 47 μmol/L, *p* = 0.046) at diagnosis. In contrast, more patients infected with HEV genotype 3 reported dark urine (71% vs. 39%, *p* = 0.02) and experienced asthenia (89% vs. 58%, *p* < 0.01) than did those infected with HEV genotype 4. Two HEV genotype 4-infected patients died of multi-organ failure, while none of the genotype 3-infected patients died (*p* = 0.035). Finally, stepwise regression analysis retained only a greater increase in ALT (odds-ratio: 1.0005, 95% confidence interval: 1.00012–1.00084) and less frequent fever (odds-ratio = 0.1244; 95% confidence interval: 0.01887–0.82020) for patients infected with HEV genotype 4. We conclude that HEV-4 infections are likely to be associated with higher ALT activity than HEV-3 infections. Additional immunological and virological studies are required to confirm these findings and better understand the influence, if any, of genotype on HEV pathophysiology.

## Introduction

Hepatitis E is the main cause of viral hepatitis worldwide. The 20 million cases that occur each year include more than 3 million symptomatic cases and 60,000 fatalities (Kamar et al., 2017). The virus is responsible for disease in both developing countries with suboptimal sanitary conditions and in industrialized countries where its transmission is mainly zoonotic (Kamar et al., 2017). The eight hepatitis E virus (HEV) genotypes (HEV-1 to HEV-8) that infect mammals include four major ones (HEV-1–4) each with distinct characteristics (Kamar et al., 2017). Smith et al. recently proposed reference sequences for HEV subtypes (HEV-1 to HEV-7), including six subtypes (1a–1f) in HEV-1, two (2a and 2b) in HEV-2, eleven (3a–3j and 3ra) in HEV-3 and nine (4a–4i) in HEV-4 (Smith et al., 2020). HEV-1 and HEV-2 infect only humans, mainly in resource-limited areas. HEV-3 and HEV-4 include zoonotic strains that infect both animals and humans (Doceul et al., 2016; Kamar et al., 2017).

The major transmission routes in industrialized countries are zoonotic. Eating contaminated food like undercooked pork products or game meat is probably the major mode of transmission (Kamar et al., 2017). Veterinarians and farmers working with pigs are more likely to have anti-HEV antibodies due to exposure to infected animals than are blood donors (Meng et al., 2002; Christensen et al., 2008). However, a contaminated environment could be another transmission route as the HEV genome and infectious virus have been found in sewage water and rivers (Clemente-Casares et al., 2003; Rutjes et al., 2009). Lastly, HEV has also been transmitted via blood and organ donations (Kamar et al., 2017).

HEV is a major cause of human disease in European Union countries. For the *European Food Safety Authority*, between 2007 and 2017 more than 21,000 HEV acute clinical cases with 28 fatalities were notified in European Union (Efsa Panel on Biological Hazards (Biohaz) et al., 2017). HEV-3 is the prevalent genotype and subtypes 3f and 3c the main infectious agents in the EU (Efsa Panel on Biological Hazards (Biohaz) et al., 2017; Izopet et al., 2019), while HEV genotype 4 is predominant in Asia, particularly China and Japan (Doceul et al., 2016; Kamar et al., 2017; Primadharsini et al., 2019). Several autochthonous cases of HEV-4 have been reported in Europe, in both humans (Wichmann et al., 2008; Colson et al., 2012a,b; Tesse et al., 2012; Garbuglia et al., 2013; Jeblaoui et al., 2013; Bouamra et al., 2014; Midgley et al., 2014) and pigs (Hakze-van der Honing et al., 2011), over the past 10 years or so. The first HEV-4 reports came from a single autochthonous case in Germany (HEV-4f) and from surveys of swine in Belgium (HEV-4b) (Wichmann et al., 2008; Hakze-van der Honing et al., 2011). In 2011, an outbreak in Italy affecting five people living in the same area with no history of traveling to areas where HEV-4 is endemic was identified as due to HEV-4d, a strain that is similar to Chinese swine isolates (Garbuglia et al., 2013). The authors suggest that autochthonous HEV-4 infections are emerging in Europe and have been transmitted by at least two distinct sources.

Hepatitis E produces a benign infection in most immunocompetent people. Classic forms can have a wide range of symptoms, including fever, nausea, anorexia, asthenia, vomiting, abdominal pain, and icterus (Kamar et al., 2017). Liver function tests are often abnormal, with signs of hepatic cytolysis (increased transaminases), cholestasis, and sometimes even liver failure. However, severe acute or fulminant hepatitis is rare, usually occurring in patients with underlying chronic liver disease and pregnant women with an HEV-1 or HEV-2 infection (Wu et al., 2020). The virus can also produce extra-hepatic symptoms like neurological disorders, renal failure, pancreatitis, and hematological disorders (Kamar et al., 2017). The biological and clinical manifestations are usually less severe in immunocompromised patients, but in few cases HEV infection can become chronic and lead to cirrhosis (Kamar et al., 2017).

A few Japanese studies (Mizuo et al., 2005; Ohnishi et al., 2006; Murata et al., 2019) and a single European one (Jeblaoui et al., 2013) have investigated the differences in the pathogenicities of genotypes 4 and 3. Some studies conducted in Japan demonstrated higher peak aminotransferase activities (Mizuo et al., 2005; Ohnishi et al., 2006; Murata et al., 2019), shorter prothrombin times (Mizuo et al., 2005; Ohnishi et al., 2006; Murata et al., 2019) and higher total bilirubin concentrations (Mizuo et al., 2005; Murata et al., 2019) in HEV-4 infected patients. A study conducted in France reported higher ALT activities and more icterus among HEV-4 infected patients (Jeblaoui et al., 2013). However, all the above-mentioned studies compared only a limited number of HEV-3/HEV-4 infected patients. This study assesses the differences in the severity of disease caused by HEV-3 and HEV-4 infections by analyzing the clinical and biological data for two groups of immunocompetent patients diagnosed with acute hepatitis E in France and Belgium.

## Materials and Methods

### Patients

Plasma samples and data from HEV IgM positive patients from French or Belgian hospitals or laboratories were sent to our reference centeres for HEV RNA detection and genotyping. We included all the immunocompetent HEV-4-infected patients reported to the Belgian and French reference centeres between January 2017 and August 2019. We collected the biological, virological and clinical data for these 27 patients, 3 Belgian and 24 French, none of whom had travelled outside France or Belgium during the 2–8 weeks before symptoms onset. Immunocompromised patients were excluded.

Each HEV-4-infected patient was matched for age (±5 years) and gender with 2 HEV-3-infected patients selected randomly from the HEV-3 immunocompetent cases reported to the French national center. We collected the clinical symptoms (asthenia, anorexia, fever—defined as a temperature ≥38°C, abdominal pain, nausea, vomiting, icterus, dark urine, discolored stools, itching) for all the patients by telephone or from their medical reports. We recorded both extrahepatic manifestations (renal failure, pancytopenia, pancreatitis) and neurological manifestations (neuralgic amyotrophy, neuropathic pain, facial paralysis). Hepatic encephalopathy was not classified as a neurological manifestation *per se* as it is linked to hepatic failure. We recorded several biological parameters, including plasma virus load, transaminase activities [alanine aminotransferase (ALT) and aspartate aminotransferase (AST)], prothrombin time and total bilirubin) at the time of diagnosis. We also looked for evidence of existing underlying liver disease (cirrhosis or steatosis). Lastly, we recorded the outcome of the hepatitis E infection: hospitalization or the patient’s death.

### Virological Methods

The frozen plasma samples collected at the Belgian and French reference centers from suspected HEV infected patients were analyzed in Toulouse or Brussels. HEV RNA was extracted from 500 μl plasma using the MagnaPure 96 instrument (France, elution volume 50 μl), or from 140 μl plasma with the EasyMag instrument (Brussels, elution volume 60 μl). The HEV RNA concentration in 25 μl aliquots of extract was measured using the RealStar HEV RNA 2.0 kit from Altona (Abravanel et al., 2013). The HEV genotype and subtype were determined by population sequencing of open reading frame 2 (ORF2) (Legrand-Abravanel et al., 2009; Suin et al., 2019) and by phylogenetic analysis based on the reference sequences proposed by Smith et al. (2020). A 345 nt fragment of ORF2 (nucleotides 5,994–6,294 of the M73218 genome) was amplified with a hemi-nested PCR protocol (Legrand-Abravanel et al., 2009; Suin et al., 2019). For phylogenetic analysis, nested RT-PCR was performed, targeting the ORF2 region (structural proteins), using primers HEV5944S (5′-GTGGCYGAGGAGGAGGCKAC-3′) and HEV-6484AS (5′-CCCTTRTCCTGCTGNGCATTCTCGACAGA-3′) in the first round and HEV-5930S (5′-CCCTTRTCCTGCTGNGCATTCT CGACAGA-3′) and HEV-6320AS (5′-TGYTGGTTRTCAT AATCCTG-3′) in the second round. The PCR products of these amplifications were purified and sequenced using the fluorescent dye terminator method for Big DyeTerminator cycle sequencing (Applied Biosystems, Paris, France) with the same primers as those used for amplification on an Applied Biosystems ABI 3130 XL analyzer. The nucleotide alignments were performed using ClustalX v2.0^1^. The tree was constructed using the MEGA v5.0 software^2^ with the neighbor-joining method. Reference sequences of avian HEV were included as an outgroup to root the tree. The final tree was prepared using the bootstrap method (bootstrapped with 1,000 replicates). The sequences have been submitted to Genbank (accession numbers: MW655496 to MW655522).

### Statistical Analysis

The anonymized data were analyzed using Stata version 14 (StataCorp LP, College Station, TX, United States). The χ^2^-test was used for categorical variables and the Mann-Whitney *U*-test or Wilcoxon rank-sum test were used to compare continuous variables. A *P*-value of < 0.05 was considered to be statistically significant. We used stepwise logistic regression analysis to identify variables independently associated with HEV-4 infections. The model takes into account all the χ^2^ or Mann-Whitney test parameters with *p*-values of < 0.20.

## Results

**FIGURE 1:**
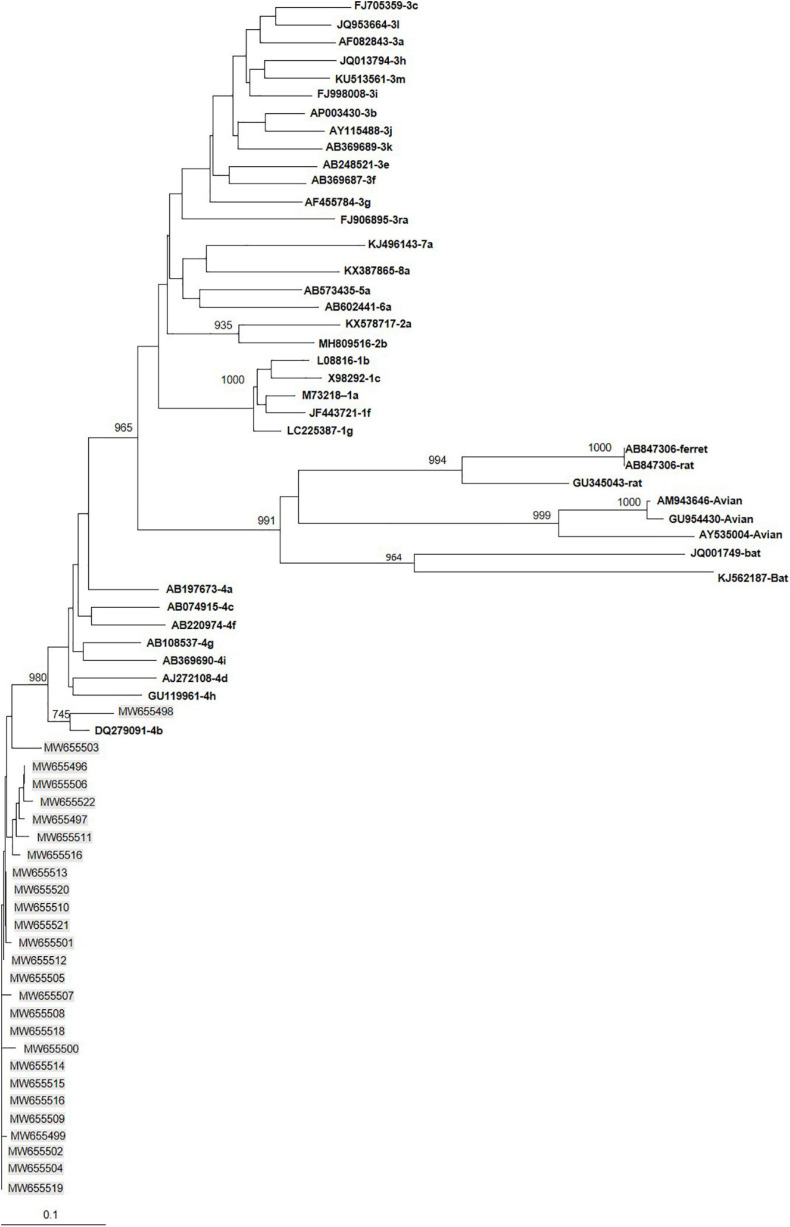
Phylogenetic tree constructed using 345-nt-long partial sequences within ORF2. Genetic distances were calculated using the Kimura two-parameter method, phylogenetic trees were plotted by the neighbor-joining method. Bootstrap values acquired after 1,000 replications are shown (branch lengths measured in the number of substitutions per site). Patients’ sequences, with the accession number highlighted in gray, were compared to the reference sequences of subtypes 3 proposed by Smith et al. (2020). Accession numbers, genotype and subtypes of the reference sequences are in bold.

All 27 immunocompetent HEV-4-infected patients were infected with subtype 4b (Figure 1). They were matched with 42 patients infected with HEV-3f, 9 with HEV-3c, and 3 with HEV-3l.

The median age of the 27 HEV-4-infected patients and 54 HEV-3-infected patients was 60 years (range 31–84 years). Their median ALT and AST activities were 1,652 (range 37–8,900 IU/L) and 1022 IU/L (range 22–11,401 IU/L); their total median bilirubin, prothrombin, and HEV RNA plasma concentrations were 68.9 μmol/L (range 3.8–446 μmol/L), 91% (range 19–100%) and 636 000 UI/mL (range 1,110–146.000.000 UI/mL), respectively.

More HEV-3-infected subjects appeared to suffer from asthenia than did those infected with genotype 4 (*p* = 0.002) and more of them also reported dark urine than did the HEV-4 patients (*p* = 0.017). There were no other significant differences in the clinical manifestations of the two groups; particularly no difference in their neurological manifestations or other extra-hepatic disorders. Two HEV-4 patients with underlying cirrhosis died of multi-organ failure, while none of the genotype 3 patients died (*p* = 0.035).

Bivariate analysis found that the HEV-4 patients had significantly higher ALT (2,067 IU/L) and AST (1,581 IU/L) activities and total bilirubin (92.4 μmol/L) concentrations than did the HEV-3 patients (ALT: 1,566 IU/L; *p* = 0.016; AST: 657 IU/L; *p* = 0.003; bilirubin: 47 μmol/L; *p* = 0.046) at diagnosis (Table 1). We found no genotype-dependent differences in existing chronic disease, prothrombin time, or virus load.

**TABLE 1:** Patient characteristics according to HEV genotype.

	**HEV-4 infected patients *N* = 27**	**HEV-3 infected patients *N* = 54**	***P* value**	**Odds-ratio**	**Standard error**	***P* > *z***	**95% Confidence interval**
Gender							
men women	18 9	36 18	1.0				
Median age in year (standard deviation)	60 (12.5)	60 (13.1)	0.9				
Existing chronic liver disease	5 (18.5%)	6 (11.1%)	0.36				
Hospitalization	22 (81.5%)	38 (71.7%)	0.34				
Asthenia	14 (58.3%)	47 (88.7%)	0.002				
Anorexia	9 (37.5%)	30 (55.6%)	0.14				
Fever> 38°C	5 (20.8%)	21 (38.9%)	0.12	0.1244	0.12	0.03	0.018-0.82
Nausea	6 (25%)	21 (38.9%)	0.23				
Vomiting	4 (16.7%)	21 (38.9%)	0.05				
Abdominal pain	13 (54.2%)	17 (31.5%)	0.06				
Mucosal icterus	16 (66.7%	27 (50%)	0.17				
Colored urine	7 (38.9%)	34 (70.8%)	0.02				
Discolored stool	6 (37.5%)	22 (45.8%)	0.561				
Pruritus	4 (16.7%	17 (31.5%)	0.17				
Extrahepatic manifestations (except neurological)	4 (15.4%)	5 (12.5%)	0.74				
Neurological manifestations (except hepatic encephalopathy)	2 (8.3%)	11 (20.4%)	0.19				
Median Virus load (UI/mL)	996 000	453 000	0.63				
Median ALT on entry (UI/L)	2067	1566	0.016	1.0005	0.0002	0.01	1.00012–1.00084
Median AST on entry (UI/L)	1581	657	0.003				
Median total bilirubin (μ mol/L) on entry	92.4	47	0.04				

Stepwise logistic regression indicated that genotype 4 infections were associated with higher ALT concentrations (OR = 1.0005; *p* = 0.01) and fewer fevers (OR = 0.1244; *p* = 0.03) than were HEV-3 infections (Table 1).

## Discussion

The evaluation of clinical and biological data of 27 cases of HEV-4 infections matched for age and gender with 54 cases of HEV-3 infections detected in Belgium and France demonstrated, based on the logistic regression analysis, that the HEV-4 infected patients had significant higher ALT activities.

HEV-4 is predominant in Asia, but this genotype has also been found in human populations in several European countries (Wichmann et al., 2008; Colson et al., 2012a,b; Tesse et al., 2012; Garbuglia et al., 2013; Jeblaoui et al., 2013; Bouamra et al., 2014; Midgley et al., 2014). Japanese studies suggest that HEV-4 infections are associated with more severe hepatitis, higher peak aminotransferase activities (Mizuo et al., 2005; Ohnishi et al., 2006; Murata et al., 2019), shorter prothrombin times (Mizuo et al., 2005; Ohnishi et al., 2006; Murata et al., 2019) and higher total bilirubin concentrations (Mizuo et al., 2005; Murata et al., 2019). A previous European study also found higher ALT activities and more icterus among HEV-4 infected patients (Jeblaoui et al., 2013). However, these studies of the two zoonotic genotypes often involved limited, unbalanced patient groups. The Japanese studies included only a few (≤ 11) HEV-3-infected patients (Mizuo et al., 2005; Ohnishi et al., 2006; Murata et al., 2019), while the French study compared 9 cases of HEV-4 with 208 cases of HEV-3 (Jeblaoui et al., 2013). The results of our bivariate and multivariate analyses agree with those of previous studies (Mizuo et al., 2005; Ohnishi et al., 2006; Jeblaoui et al., 2013; Murata et al., 2019): HEV-4 infections were associated with higher ALT activities. These patients also suffered fewer episodes of fever than did patients with HEV-3 infections; this clinical manifestation was not investigated in previous Japanese and French studies. Bivariate analysis also indicated that HEV-4 infected-patients had higher AST activities and greater total bilirubin concentrations. Surprisingly, more of the HEV-3-infected patients reported producing dark urine and suffered from asthenia than did the HEV-4-infected patients. The numbers of severe hepatitis cases (defined as a prothrombin level < 50%) in the two groups were similar. However, two deaths were recorded among HEV-4 infected patients while none among HEV-3 infected patients. This observation, although statistically significant, was not confirmed by the multivariate analysis, probably because of the small number of deaths recorded. It is always possible that the deaths of our genotype-4-infected patients were linked to other comorbidities that were not recorded by our questionnaire. In this regard, no difference in the mortality rate was reported in a study performing a systematic review and pooled analysis of acute liver failure caused by HEV-3 and HEV-4 (Haffar et al., 2018). Further studies are needed to determine whether genotype can influence the mortality rate in HEV infected patients in industrialized countries.

We previously reported that different HEV-3 subtypes could influence the clinical and biological features of an HEV infection (Subissi et al., 2019; Abravanel et al., 2020). In this study, immunocompetent patients infected with HEV subtype 3f suffered more from fever, had higher virus loads and required hospitalization than did those with subtype 3c infections (Abravanel et al., 2020). All the genotype 4-infected patients in the present study were infected with subtype 4b. Therefore, the influence of the HEV-4 subtype was not investigated.

Interestingly, we found that HEV-3-infected patients were more likely to have a fever than HEV-4 infected patients. This observation, although possibly influenced by many other factors, might be due to genotype-linked differences in the innate and inflammatory responses. Indeed, several studies reported HEV genotype-dependent stimulation of immune mediators (Syedbasha and Egli, 2017; Gouilly et al., 2018; Murata et al., 2019), possibly leading to differences in pathogenicity.

The main limitations of our study concern data that were not collected, such as the influence of patient comorbidities. The severity of an infection could be altered by conditions like obesity or diabetes and we were unable to assess the histological severity of the liver disease, as liver biopsies were not taken. Another limitation is a lack of information on the possible sources of infection.

## Conclusion

We conclude that HEV-4 infections are likely to be associated with higher ALT activity than HEV-3 infections. Additional immunological and virological studies are now required to fully describe the pathophysiology of the main HEV genotypes.

## Data Availability Statement

The raw data supporting the conclusions of this article will be made available by the authors, without undue reservation.

## Ethics Statement

Ethical approval was not provided for this study on human participants because This was a non-interventional study. Biological material and clinical data were obtained only via standard viral diagnostics following a physician’s order (no supplemental or modified sampling). Data were analyzed anonymously. The French Public Health law (CSP Art L 1121-1.1) does not require written informed consent from the patients for such a protocol. Written informed consent was not provided because this was a non-interventional study. Biological material and clinical data were obtained only via standard viral diagnostics following a physician’s order (no supplemental or modified sampling). Data were analyzed anonymously. The French Public Health law (CSP Art L 1121-1.1) does not require written informed consent from the patients for such a protocol.

## Author Contributions

JI, FM, and FA: drafting and refining the manuscript. VS, SL, and TV: critical reading of the manuscript. CD statistical analyses. FM, VS, J-MP, CS, ET, TV, LB, and SL: collected the data. All authors have read and approved the manuscript, contributed to the article and approved the submitted version.

## Conflict of Interest

The authors declare that the research was conducted in the absence of any commercial or financial relationships that could be construed as a potential conflict of interest.

## Footnotes

1 http://www.clustal.org/download/current/

2 http://www.megasoftware.net/

## Abbreviations

ALT: alanine aminotransferase
AST: aspartate aminotransferase
HEV: hepatitis E virus
IFN- λ 3: interferon lambda 3
RNA: ribonucleic acid.
